# Analysis of road traffic injuries and casualties in China: a ten-year nationwide longitudinal study

**DOI:** 10.7717/peerj.14046

**Published:** 2022-09-15

**Authors:** Miao Qi, Xiuli Hu, Xiahong Li, Xue Wang, Xiuquan Shi

**Affiliations:** 1 Department of Epidemiology and Health Statistics, School of Public Health, Zunyi Medical University, Zunyi, Guizhou, China; 2 Center for Injury Research and Policy & Center for Pediatric Trauma Research, The Research Institute at Nationwide Children’s Hospital, The Ohio State University College of Medicine, Columbus, OH, USA

**Keywords:** RTIs, Traffic accidents, Road safety, Casualties, Temporal trend, Risk factors

## Abstract

**Background:**

Road traffic injuries (RTIs) are a serious global problem, and a huge challenge for both economic development and public health.

**Methods:**

This longitudinal study was based on the national data from Chinese authorities. Descriptive analysis was utilized to analyze the prevalence and trend of RTIs among different types, groups and regions. In addition, ridge regression or/and curve regression were also used to explore the relationship between those possible influencing factors and RTIs.

**Results:**

From 2010 to 2019, the death toll from motor vehicle accidents (MVAs) decreased firstly and then increased, while the death toll from non-MVAs continued to rise since 2012, and the death toll from pedestrian and occupant accidents fluctuated only a little. The mortality rate of MVA was relatively stable from 2010 to 2012, and declined from 2013. The mortality rate of motor vehicle accidents was higher in rural than urban, the same to male compared with female. The results of ridge regression showed that gross domestic product (GDP)-per-capita, total population, number of health personnel and car ownership were positively correlated with the death toll of non-MVAs (*P* < 0.05). Additionally, the results of curve regression suggested that the quadratic or cubic relationship between each factor and the number of MVAs was well fitted, while only partially fitted in fatalities.

**Conclusions:**

In recent years, RTIs in China show different trends, and the problem of non-motor vehicle traffic injuries has been neglected which should be paid more attention. Moreover, according to the new trends and traffic conditions in RTIs revealed in this study, it is necessary to formulate targeted intervention measures establish a multi-faceted comprehensive safety system to reduce the disease burden of RTIs as well as the total injuries.

## Introduction

Road traffic injuries (RTIs) are a serious global human health problem and pose a huge challenge to both economic development and public health. According to the predictive report of the World Health Organization, approximately 1.35 million people die each year as a result of road traffic crashes. If the increasing trend in RTIs continues at the current speed and without urgent action, traffic deaths will become the fifth leading cause of death in the world by 2030 (World Health Organization, 2018; United Nations Secretary-General, 2012).

In the past two decades, China has implemented many intervention measures to reduce the worse road traffic accidents, such as improving traffic laws and regulations, punishing illegal behaviors, limiting speed, standardizing warning signs, raising driving license requirements, and strengthening traffic safety publicity and education (Hu et al., 2008; Research Institute of Highway Ministry of Transport, 2007). However, RTIs are still an important public health problem in China and are the primary cause of death because of injuries (Shao et al., 2018). China is the most populous developing country in the world. Motorization has increased rapidly as a result of the high-speed development of economy, which gave rise to a serious of problems. Zhang et al. (2013) estimated that there are about 55,000 newly registered motor vehicles in China every day. The number of motor vehicles nationwide was increased from 207 million in 2010 to 348 million in 2019, with an increase of 68.12% in 10 years. In addition, the length of expressways was increased from 74,100 to 149,600 km between 2010 and 2019, with an increase of 101.89%.

At the beginning of 2009, China became the largest automobile market in the world, and the total sales volume of automobiles surpassed that of the United States for the first time (Overholt, 2010). The increasing number of motor vehicles is one of the main factors leading to road traffic deaths. RTIs are more hazards to people aged 5–29 and over 65, men, and rural residents, in addition, pedestrians, cyclists and motorcyclists accounted for more than half of all deaths in the world, which were vulnerable road users (World Health Organization, 2018). Tan et al. (2014) reported that mortality number of deaths and Years of Life Lost (YLLs) from RTIs were predicted to decrease slightly from 2015 to 2030.

Although some countries had achieved great success in reducing road traffic deaths, the risk of road traffic deaths was still much higher in low- and middle-income countries than that in high-income countries (World Health Organization, 2018), which suggested there might be a strong correlation between the risk of road traffic deaths and the country’s income levels. The main reason of less deaths in high-income countries might be there were more prevention researches, intervention measures and policy management in high-income countries than those in low- and middle-income countries (Pal et al., 2018). Effective intervention strategies have been taken out to prevent or reduce RTIs to a large extent in national practices (World Health Organization, 2018), while these experience may not be applied to other countries due to the differences in economic factors, road conditions, policies and regulations.

There are a large territory, high population density, increasing motorization, widespread road traffic accidents and low effective interventions in China. In recent years (especially in 2016 and 2017), the government of China has attached great importance to road safety, and put forward the future road safety goals in many policy documents, such as the “13th Five-Year Plan” for road safety (*e.g.*, Production Safety Commission of the State Council, 2017). Based on socio-demographic factors, geographical areas and different road types, epidemiological characteristics, the trends and main patterns of RTIs in China have been reported (Wang et al., 2019; Zhang et al., 2011). Previous studies have shown that various economic factors, population size, road length, and car ownership have an impact on road traffic casualties and/or MVAs deaths (Harper, Charters & Strumpf, 2015; Wang et al., 2012), while road type, cyclist behavior characteristics (including distraction driving, not wearing a helmet, riding in the wrong direction, violating traffic signals) have an effect on non-MVAs casualties (Pai et al., 2018; Zhang et al., 2018; Zhong et al., 2022). To our knowledge, there were not sufficient researches on motor vehicle accident injuries in national road traffics in China, especially non-motor vehicle accidents and pedestrian injuries. Considered the rapid development of the society and the constant changes in traffic dynamics, the epidemic trend and influencing factors of RTIs in recent years might have altered based on the latest statistical data, thus it is urgent to develop solutions of RTI, the major public problem in China.

In this study, we hypothesized that RTIs in China had peculiar characteristics and influential factors which could make RTIs decrease year by year. Therefore, this study will report on road traffic accidents in China in recent 10 years, aiming to reveal the development trend and possible influencing factors of RTIs, especially MVAs and non-MVAs which show different characteristics. We hope that the results of our study can increase more attention to non-MVA which is easy to be ignored and provide pieces of evidence and useful preventive suggestions for future road traffic safety decision-makers in China and other similar countries.

## Materials and Methods

### Data source

This longitudinal study was based on the national data in China. The data were extracted from the National Bureau of Statistics of China (2010-2019), the Ministry of Public Security of the People’s Republic of China (2010-2019) and the Ministry of Transport of the People’s Republic of China (2010-2019). The information included the basic characteristics of RTIs, such as the number of traffic accidents, death toll, injuries and direct property losses. Data of GDP-per-capita, total population, number of health personnel, car ownership, number of motor vehicle drivers and highway mileage were all obtained from the annual statistical bulletin and statistical yearbook, and the traffic injury mortality rate of urban and rural residents was obtained from the Ministry of Health Registration (MOH-VR) comprehensive data published in the China Health Statistics Yearbook (National Health Commission of the People’s Republic of China, 2011-2020), which also consisted of the mortality rate among sex and diseases. Data for Hong Kong, Macau, and Taiwan were not consisted in the analysis due to the lack of some detailed data.

It is worth noting that the unified “National Road Traffic Accident Information System” is used for the statistics of road traffic accidents in China, which covers (1) Accidents causing deaths; (2) Accidents causing serious injuries or minor injuries; (3) Property loss accidents handled by the rule procedures (Traffic Administration of the Ministry of Public Security of China, 2008). The death caused by a road traffic accident must be confirmed by first aid and medical personnel, and a death certificate issued by a medical institution (if the injured person dies 7 days after the accident, the death toll shall not be included in the scope of the death toll statistics) (Traffic Administration of the Ministry of Public Security of China, 2018).

According to the main responsibility for accidents, road traffic accidents can be divided into motor vehicle accidents (MVAs), non-MVAs, pedestrian accidents, and passenger accidents. The main analytical indicators used in this research are explained as follows: (a) MVAs includes car accidents, motorcycle accidents, tram accidents, tractor accidents, special mechanical accidents, agricultural transport vehicle accidents, *etc.* (b) Non-MVAs consists rickshaw accidents, animal car accidents, bicycle accidents, *etc.* (c) Pedestrian and/or passenger accidents refers to accidents in which pedestrians and/or passengers are primarily responsible.

### Statistical analysis

Descriptive epidemiology was used to describe the prevalence and trends of RTIs. The curve regression analysis was applied to analyze the relationships between the number of motor vehicle accidents (MVAs), death toll and GDP-per-capita, number of health personnel, car ownership, number of motor vehicle drivers, and highway mileage. And multiple linear regression analysis was used to explore the relationship between the number of non-motor vehicle deaths and various factors including GDP-per-capita, total population, number of health personnel, and car ownership, number of motor vehicle drivers, and highway mileage. However, the correlation between the above six characteristic parameters is strong, which may be not suitable for all modeling. Due to improving the model estimation accuracy, we use the stepwise regression method to exclude two characteristic parameters and build an optimal model (but all variance inflation factor, VIF > 10). Thus we conducted the ridge regression method (Hoerl & Kennard, 1970) to solve the collinearity problem. This article adopts the method of ridge trace plot, When the ridge trace of the characteristic parameter tends to be stable, the undetermined coefficient of the characteristic parameters were calculated according to the ridge trace parameter *k* (the ridge regression coefficient), and the estimation model was provided.

All tests were two-tailed, and *P* < 0.05 was considered statistically significant. Database was built by using Microsoft Office Excel (version 2010) and SPSS (version 18.0, IBM Corp., Armonk, NY, USA) and macroprogram “Ridge Regression” were used for data analysis.

## Results

From 2010 to 2019, it was growing steadily in population, economy, health input and transportation construction in China. Table 1 shows the changes of GDP-per-capita, health personnel, car ownership, total population, motor vehicle drivers, and highway mileage in China in recent 10 years.

**Table 1:** Relevant variables of social population and economy in China from 2010 to 2019.

Year	GDP-per-capita (10^3^ RMB Yuan)	Health personnel (10^4^ persons)	Car ownership (10^6^ vehicles)	Total population (10^7^ persons)	Motor vehicle drivers (10^6^ persons)	Highway mileage (10^4^ miles)
2010	30.81	820.75	78.02	134.09	200.68	400.82
2011	36.30	861.60	93.56	134.74	228.18	410.64
2012	39.87	911.57	109.33	135.40	252.51	423.75
2013	43.68	979.05	126.70	136.07	269.56	435.62
2014	47.17	1,023.42	145.98	136.78	298.92	446.39
2015	50.24	1,069.39	162.84	137.46	328.53	457.73
2016	54.14	1,117.29	185.75	138.27	358.77	469.63
2017	60.01	1,174.90	209.07	139.01	360.17	477.35
2018	66.01	1,230.03	232.31	139.54	410.30	484.65
2019	70.89	1,292.83	253.76	140.01	436.37	501.25
Total	499.12	10,480.83	1,597.32	1,371.37	3,143.99	4,507.83

### Epidemic characteristics and trends of RTIs

The basic features of road traffic accidents in China from 2010 to 2019 included a total of 2,125,994 traffic accidents, resulting in 2,292,026 injuries, 615,515 deaths, and direct property loss of 11,482.74 million RMB.

**Figure 1:**
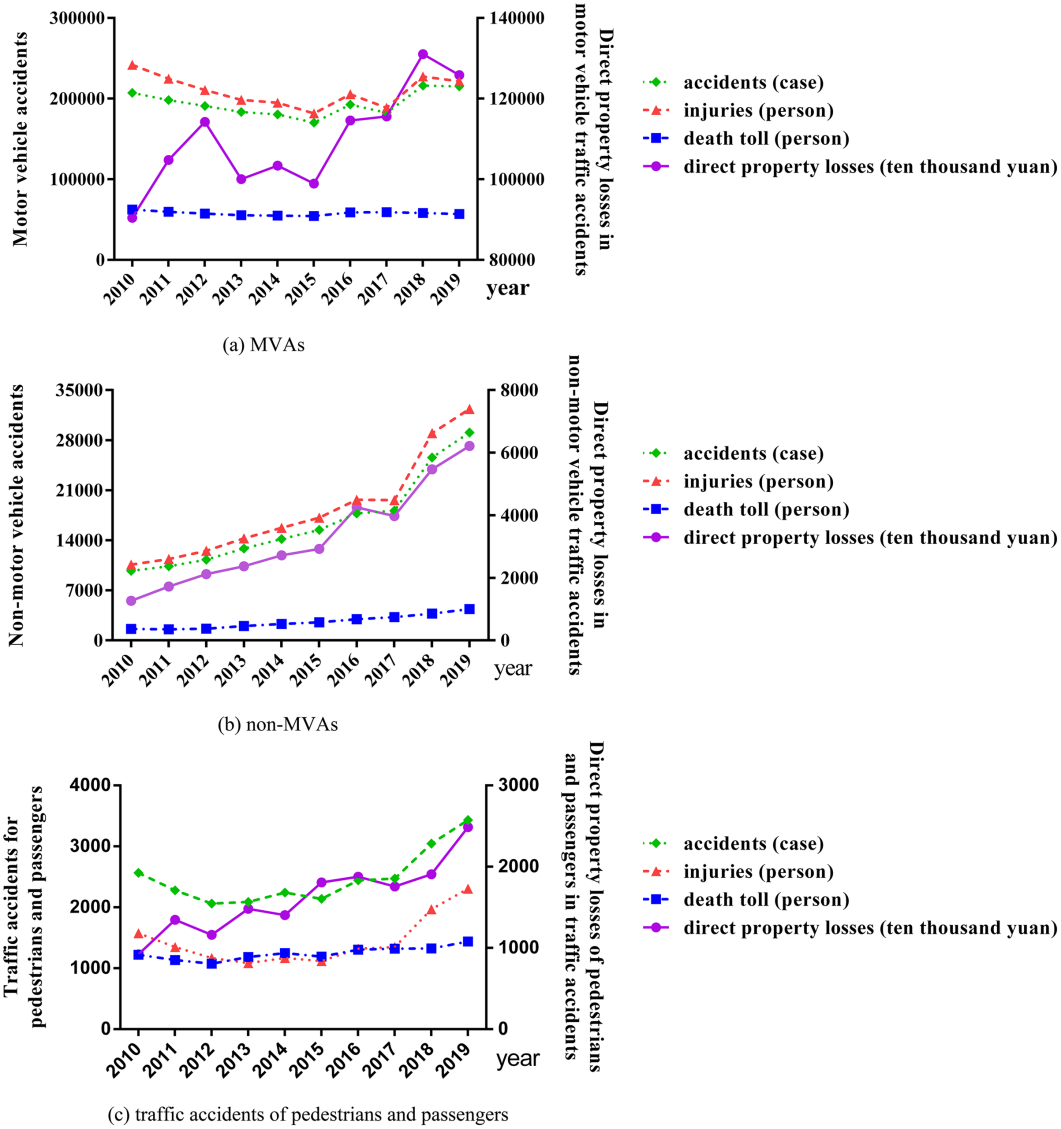
The trend of MVAs, non-MVAs, and traffic accidents of pedestrians and passengers in China from 2010 to 2019.

From 2010 to 2015, the number of MVAs, injuries and deaths declined steadily, while the deaths rose in 2016 and 2017 and declined again in 2018 and 2019. The number of incidents showed the similar trend which is that the injured people rose in 2016 and then fell in 2017, while rose again in 2018 and then fell in 2019 (still higher than that in 2015) (see Fig. 1A).

From 2010 to 2019, the number of non-MVAs and injuries had always kept rising rapidly, while declined a little in 2017. The deaths have been on an upward trend, while it decreased slightly in 2011 (see Fig. 1B).

The number of traffic accidents, injuries and deaths among pedestrians and passengers fluctuated up and down in a certain range from 2010 to 2019. While the number of accidents increased by more than 20% and injuries increased by more than 40% between 2017 and 2018 (see Fig. 1C).

**Figure 2:**
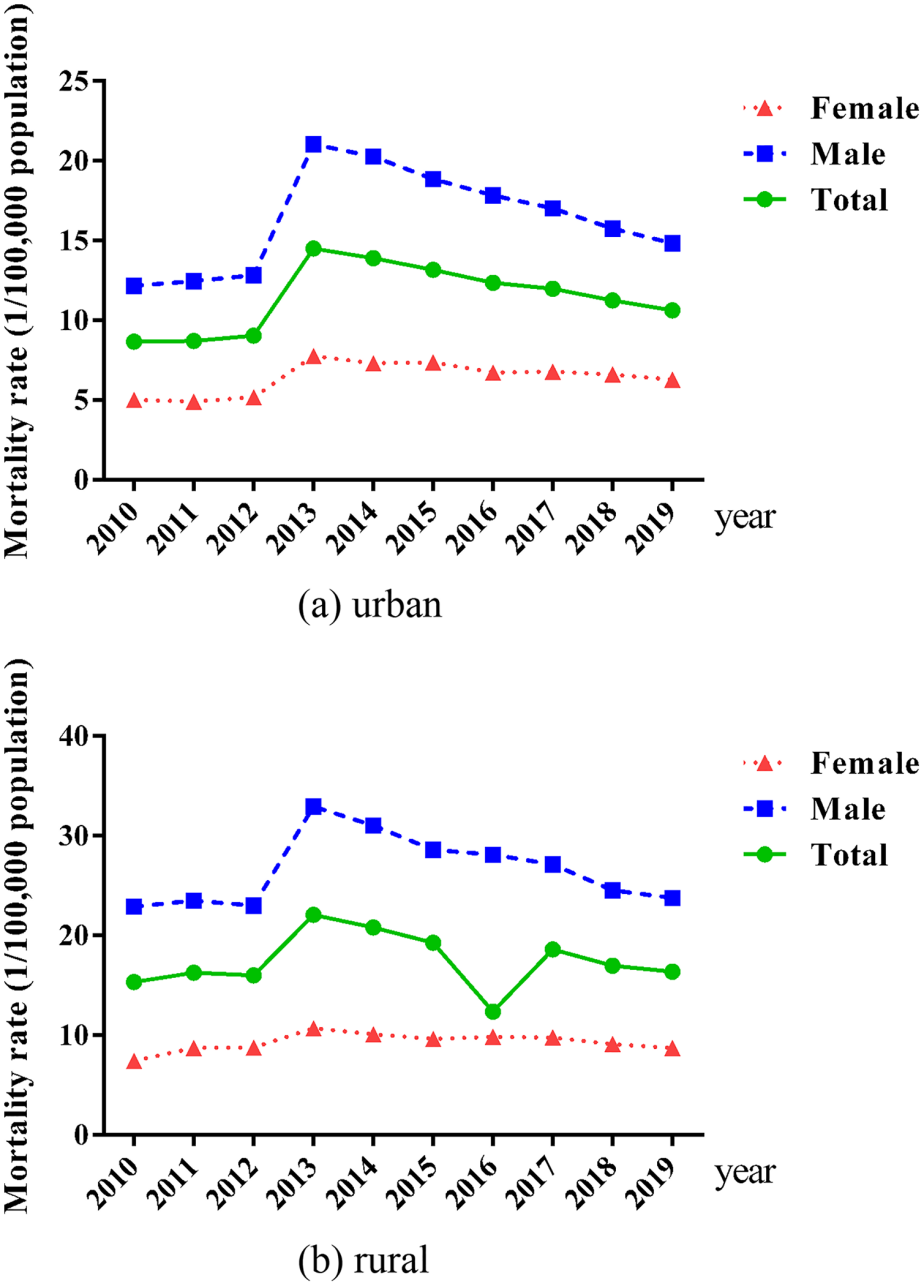
Trends in the mortality rate of urban/rural residents from MVAs from 2010 to 2019. (A) Urban. (B) Rural.

In recent 10 years, the fatality rate of motor vehicle traffic in urban and rural areas as well as males and females showed the similar change trend. From 2010 to 2012, it was relatively stable, but after a rapid rose in 2013, it continued to decline, especially for males. The MVAs mortality rate was higher in rural than urban, the same to males compared with females (see Fig. 2).

### Analysis of influencing factors of MVAs

The relationships between the number of MVAs, deaths (dependent variable) and GDP-per-capita, number of health personnel, car ownership, number of motor vehicle drivers, and highway mileage (independent variables) were analyzed by curve regression. There was a non-linear relationship between the dependent variable and the independent variables (see Table 2).

**Table 2:** Correlation coefficient between variables.

Variables	1	2	3	4	5	6	7	8	9	10
1 The number of MVAs (cases)	1	0.552	0.494	0.386	0.275	0.189	0.221	0.272	0.258	0.173
2 MVAs deaths (persons)		1	−0.176	−0.161	−0.281	−0.287	−0.304	−0.248	−0.297	−0.330
3 The number of non-MVAs (cases)			1	0.981*	0.962*	0.928*	0.948*	0.958*	0.958*	0.931*
4 non-MVAs deaths (persons)				1	0.981*	0.968*	0.979*	0.986*	0.977*	0.968*
5 GDP-per-capita (10^3^ RMB Yuan)					1	0.991*	0.996*	0.998*	0.992*	0.990*
6 Total population (10^7^ persons)						1	0.997*	0.995*	0.992*	0.997*
7 Health personnel (10^4^ persons)							1	0.998*	0.994*	0.997*
8 Car ownership (10^6^ vehicles)								1	0.994*	0.993*
9 Motor vehicle drivers (10^6^ persons)									1	0.993*
10 Highway mileage (10^4^ km)										1

**Note:**

* *P* < 0.01.

The quadratic or cubic curve regression model was built by each factor and the number of MVAs, in which the minimum determination coefficient was R_min_^2^ = 0.72, F_min_ = 9.1, *P*_max_ < 0.05, indicating that the regression models was fitting well (see Table 3). Though the quadratic or cubic curve regression model were constructed by the same factors and the number of MVAs deaths, only the curve regression model constructed by car ownership showed statistical significance and presented a cubic model. The specific R^2^, F value and *P* value of all curve regression fitting models were shown in Table 4.

**Table 3:** Curve regression fitting model of various factors and the number of MVAs.

Variables name	Curve regression model equation	R_1_^2^	F_1_ value	*P*_*1*_ value
GDP-per-capita (10^3^ RMB Yuan)	385,452.06 − 8,319.05x + 84.52x^2^	0.78	12.56	0.005
Health personnel (10^4^ persons)	835,551.46 − 1,267.54x + 0.61x^2^	0.78	12.12	0.005
Car ownership (10^6^ vehicles)	290,249.09 − 1,411.85x + 4.49x^2^	0.80	13.75	0.004
Motor vehicle drivers (10^6^ persons)	424,255.23 − 1,587.24x + 2.57x^2^	0.85	19.15	0.001
Highway mileage (10^4^ km)	2,892,528.56 − 12,141.31x + 13.59x^2^	0.72	9.10	0.011

**Table 4:** Curve regression fitting model of various factors and MVAs deaths.

Variables name	Curve regression model equation	R_2_^2^	F_2_ value	*P*_*2*_ value
GDP-per-capita (10^3^ RMB Yuan)	84,441.61 − 1,053.85x + 9.78x^2^	0.45	2.82	0.126
Health personnel (10^4^ persons)	140,110.26 − 154.97 x + 0.07x^2^	0.48	3.18	0.104
Car ownership (10^6^ vehicles)	115,523.75 − 1,111.32x + 6.57x^2^ − 0.01x^3^	0.88	14.26	0.004
Motor vehicle drivers (10^6^ persons)	87,181.53 − 185.64x + 0.28x^2^	0.44	2.73	0.133
Highway mileage (10^4^ km)	403,752.15 − 1,521.40x + 1.66x^2^	0.52	3.67	0.081

### Analysis of influencing factors of non-MVAs

There was statistically significant (*P* < 0.001) in Pearson correlation between GDP-per-capita, total population, health personnel, car ownership (independent variable) and non-MVAs deaths (dependent variable), and the coefficients were 0.981, 0.968, 0.979 and 0.986, respectively. Due to the linear correlation trend, a multiple linear regression model was established according to the joint influence of the above factors, and the regression coefficients of the independent variables were shown in Table 5. The correlation coefficient R^2^ of the model regression was 0.999, and the adjusted correlation coefficient R^2^_Adj_ was 0.999, which indicated that the fitting accuracy of the model was high. The F = 2,011.404 and *P* < 0.001 showed that the ordinary least squares (OLS) regression equation was significantly established. However, the results in Table 2 showed that there was a high correlation between these independent variables. The results in Table 5 displayed that the VIF of *X*_1_, *X*_2_, *X*_3_ and *X*_4_ were far greater than 10, indicating that there was serious multicollinearity between independent variables, which makes the obtained regression coefficients interpretive distortion.

**Table 5:** Regression coefficient values of some factors and non-MVAs deaths (10^2^ persons).

Variables name	Symbol	B	Standard error	Standardized coefficient	t	*P* value	VIF
(constant)	—	1,281.42	93.91	—	13.65	<0.001	
GDP-per-capita (10^3^ RMB Yuan)	X_1_	−1.14	0.14	−1.53	−8.17	<0.001	281.61
Health personnel (10^4^ persons)	X_2_	0.06	0.01	0.99	4.42	0.007	404.11
Car ownership (10^6^ vehicles)	X_3_	0.59	0.38	3.61	15.48	<0.001	437.44
Total population (10^7^ persons)	X_4_	−9.89	0.75	−2.10	−13.22	<0.001	202.67

**Figure 3:**
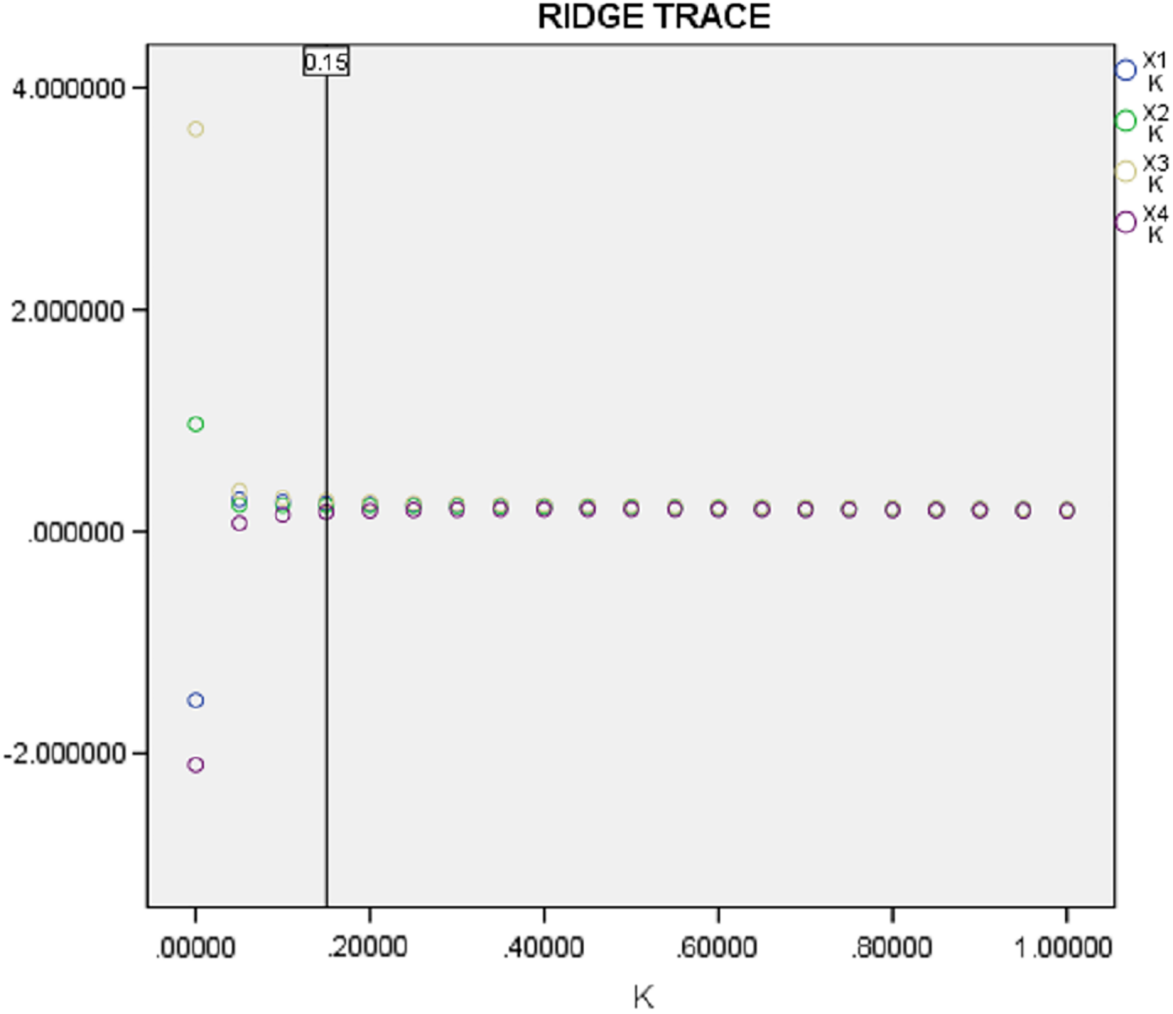
Ridge traces for each factor.

**Figure 4:**
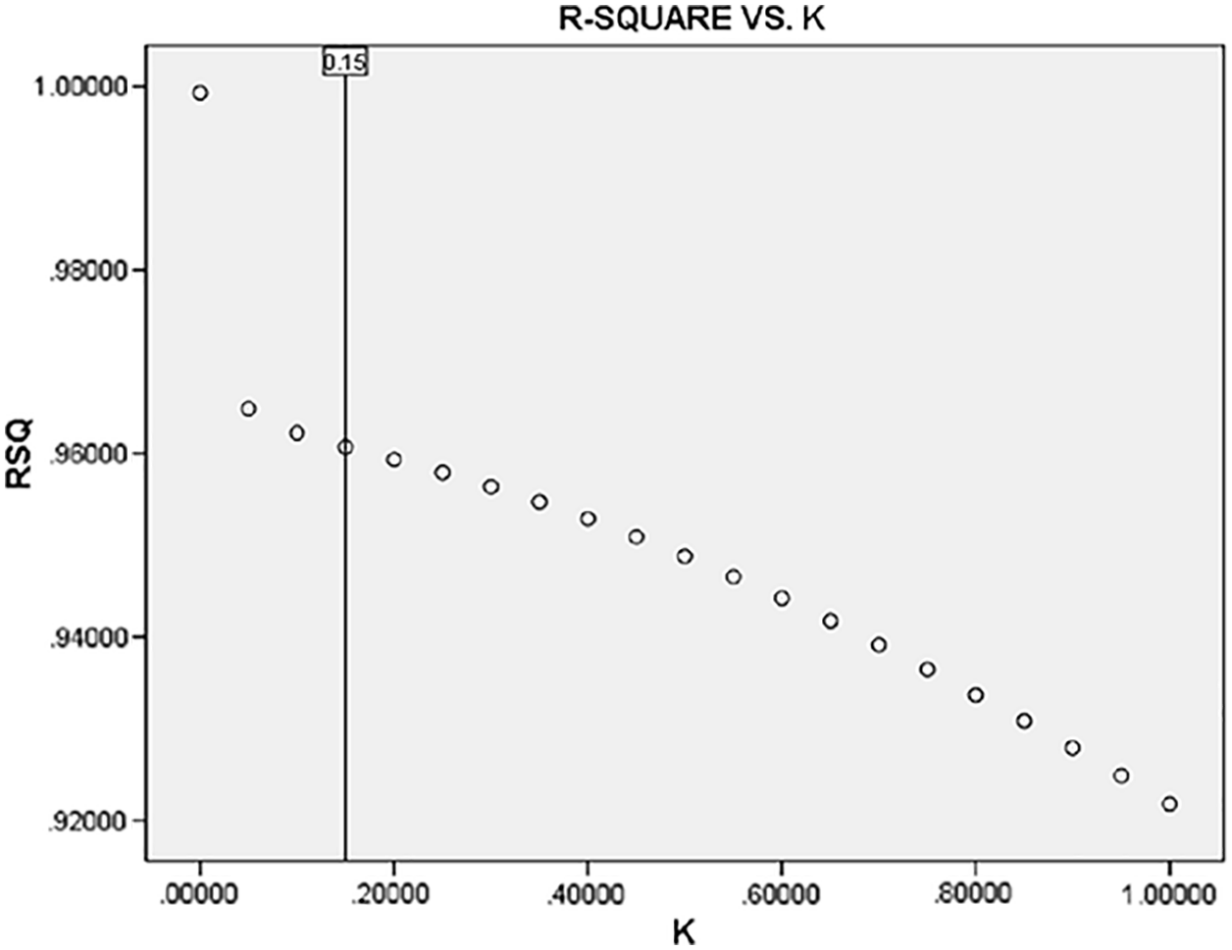
Changes of k under different RSQ.

Ridge regression was used to solve multicollinearity problems. When establishing the ridge regression model, the specified deviation coefficient is generally selected according to the *k-β* plot (ridge plot) (see Fig. 3). As the results show in Fig. 4, the regression line coefficients of the respective variables decrease rapidly with the increase of the *k* value. When *k* value was 0.15, the regression coefficients of the respective variables are basically stable. Based on the R^2^-*k* plot (see Fig. 4), the R^2^ of ridge regression has a low change rate and tends to be stable after *k* = 0.15. Therefore, considering better interpretability, the optimal ridge regression parameter was *k* = 0.15. Then *k* = 0.15 was added into the program, and the regression parameters were shown in Table 6. The R^2^ and R^2^_Adj_ of the model were 0.961 and 0.929, respectively. Compared with the least square regression, R^2^_Adj_ decreased, mainly because ridge regression abandoned some information in order to solve collinearity, resulting in a decrease in fitting accuracy, however, the model was still highly reliable for a small decrease. F = 30.566 and *P* = 0.001, which indicated that the ridge regression equation was significantly established.

**Table 6:** Coefficients of ridge regression.

Variables name	Symbol	B	Standard error	Standardized coefficient	t
(constant)	—	−119.272	29.817	—	−4.000
GDP-per-capita (10^3^ RMB Yuan)	X_1_	0.191	0.032	0.256	5.985
Health personnel (10^4^ persons)	X_2_	0.014	0.002	0.234	7.525
Car ownership (10^6^ vehicles)	X_3_	0.046	0.005	0.280	8.992
Total population (10^7^ persons)	X_4_	0.826	0.216	0.175	3.832

The equation of ridge regression model was as follows:

${Y}={-119.272 + 0.191{X_1} + 0.014{X_2} + 0.046{X_3} + 0.826{X_4}}$

**Figure 5:**
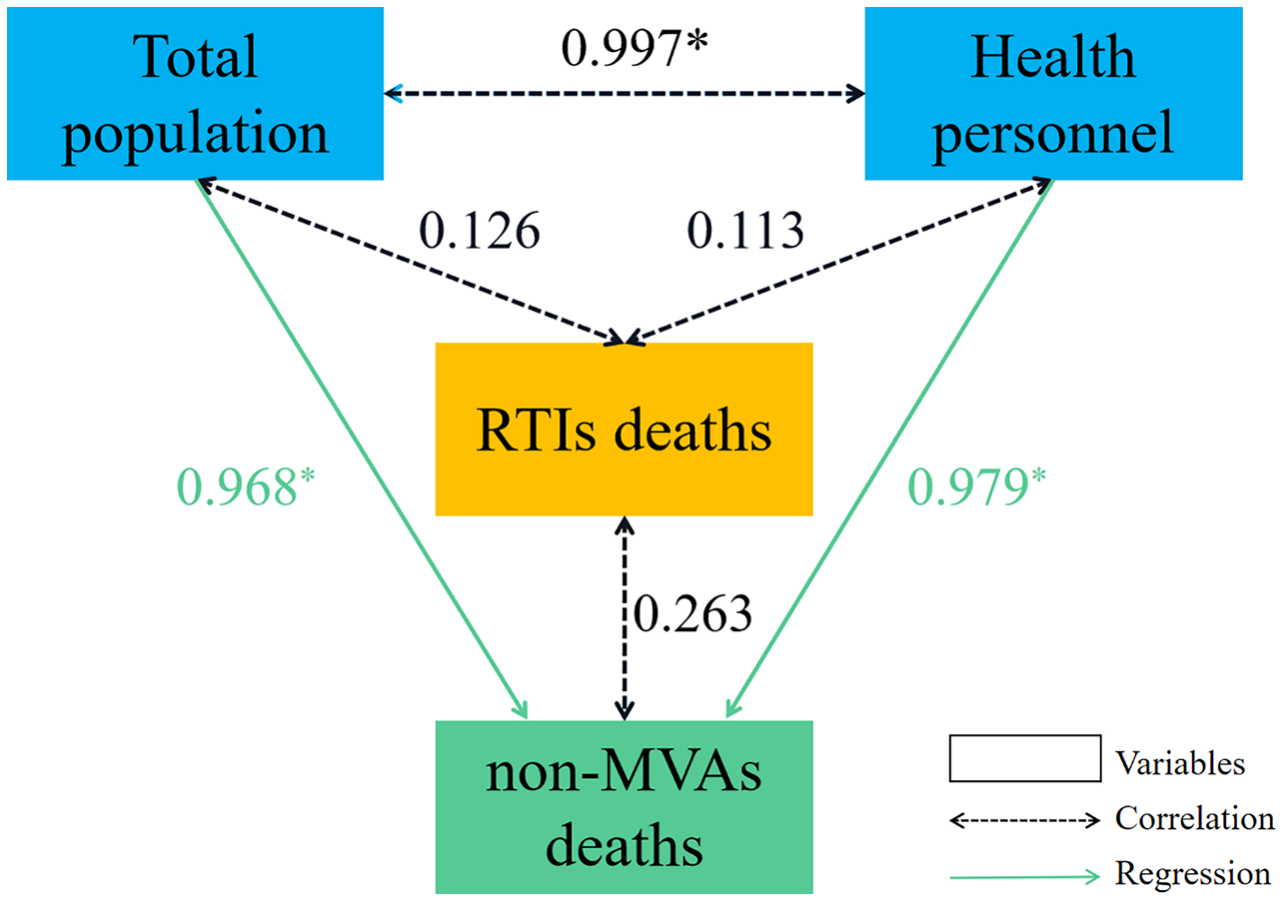
Relationship between traffic accident deaths and important population variables. Note: *P < 0.001. Total population/10^7^ persons, health population/10^4^ persons, RTIs deaths (total deaths from road traffic injuries)/10^2^ persons, non-MVAs deaths (non-motor vehicle accidents deaths)/10^2^ persons. Dashed double arrows indicate the Pearson correlation between the variables, Solid-line single arrows indicate a simple linear regression of the variables (standardization coefficient).

In the regression model, *Y* was non-MVAs deaths/10^2^ persons, *X*_1_ was GDP-per-capita/10^3^ RMB Yuan, *X*_2_ was health personnel/10^4^ persons, *X*_3_ was car ownership/10^6^ vehicles, *X*_4_ was total population/10^7^ persons. In addition, the relationship between some important variables and traffic accident deaths could be found in Fig. 5.

## Discussion

The frequency of MVAs in China continued to decrease from 2010 to 2015, which was similar to the same trend in Uganda (Vaca et al., 2020). And the reason might be due to the support provided by the national government, the increasing health and traffic inputs, and the proportion of investment in network system management, which all have an important impact on reducing the RTIs (Sun et al., 2019a). However, the frequency of MVAs fluctuated greatly from 2015 to 2019, and the overall trend was upward, which might be attributed to that a large number of three-dimensional crossings have been built in metropolitan areas for the complexity of the road traffic accident problem, and the continuous progress of urbanization, which greatly gave rise to the mobility of traffic. As an important configuration of interchange, interleaved areas are likely to increase the frequency of traffic accidents, and cause serious casualties and significant economic losses (Mao et al., 2019). New driver and vehicle numbers showed an increasing trend, the authority data showed there were 33.52 million motor vehicles registered in 2017, which resulted in a serious traffic safety situation (Wang et al., 2020).

Most foregoing studies focused on the traffic safety of motor vehicles, while they paid little attention to non-motor vehicle traffic safety. The results showed that the frequency of non-MVAs increased since 2011, which is consistent with the trend in Uganda (Vaca et al., 2020). This indicated that effective intervention measures should be carried out to solve the safety problem of non-MVAs in China. Considered economy, region, culture and other factors, non-motor vehicles still play a crucial role in the road traffic environment in China. Reasons for the increasing non-MVAs may be listed as follows: Firstly, there were poor self-protection performance and worse stability of non-motor vehicles; the size of electric bicycles, tricycles and other non-motor vehicles for the disabled is small while the speed is relatively fast. If the drivers drove illegally, they were vulnerable to be injured (Ma et al., 2019). Secondly, in consideration of the rapid progress of urbanization, there were more traffic congestion; Moreover, with the rapid development of take-out and express industries, the utilization frequency of e-bikes have been increased (Chen et al., 2019), making the risk of accidents increased. Thirdly, there were less control measures of non-motor vehicles. People who use non-motor vehicles do not need a driving license, as a result there were more illegal problems, such as speeding, running red lights, not wearing safety helmets, and reforming non-motor vehicles without permission, which greatly increased the probability of traffic injuries (Ma et al., 2019; Yang et al., 2014). Finally, road traffic safety could be interfered by cyclist distraction, weather conditions, and the physical elements of the road (Useche et al., 2018).

In addition, according to the China Health Statistics Yearbook, the mortality rate of males is higher than that of females, which is the same as previous studies (Barr et al., 2015). To some extent, the higher risk of death in road traffic accidents among men may mainly reflect the higher risk-taking behaviors among them, including distracted driving by using mobile phones (Barr et al., 2015), driving without helmets and seat belts (Riesch et al., 2013), and driving under the influence of alcohol (Asbridge et al., 2010). The higher risk of rural residents than that of urban residents may be due to worse education, more illegal and high-risk behaviors, coupled with less injury prevention and medical care in rural areas (Schreiber et al., 2017). There was same change trends of motor vehicle traffic fatality rates in urban, rural, males and females, and the death rate of MVAs was at its peak in 2013. The trend of total mortality was similar with that of road traffic collisions in Nigeria (Awoniyi et al., 2021).

RTIs are affected by many factors such as economy and society. Some potential factors of RTIs were included in the independent variable, and this study found that the number of MVAs and deaths from 2010 to 2019 were not linearly correlated with these factors, but showed a quadratic or cubic curve correlation between them, which is consistent with the non-linear relationship between economic growth and the total number of road traffic deaths in a previous research (Van Beeck, Borsboom & Mackenbach, 2000). However, this study did not show an inverted U-shaped Kuznets (1955) curve. Additionally, there were no statistical significance found in the quadratic regression model constructed by the number of MVAs deaths and influence factors, and the curve did not fit well. The possible reason was that if the traffic accident damage reached a high level in the early stage, the government would adopt a series of measures for the prevention of road traffic accidents, so the MVAs deaths declined steadily from 2010 to 2015. However, with the rapid development of urbanization and motorization as well as the rapid increase in road construction, roads are becoming more complicated than before. Therefore, the previous preventive measures cannot deal with the new complicated road traffic safety problems effectively. In some ways, the results of this study suggest that it is urge for the government to formulate new policies and targeted intervention measures to reduce RTIs under the new environment and conditions.

In addition, GDP-per-capita, total population, number of health personnel, and car ownership were significantly correlated with the number of non-MVAs deaths. Which is consistent with the research results of Sun et al. (2019b). The increasing population and cars would increase the number of trips, as a result, there were more pressure and complexity of traffic safety, higher probability of traffic accidents and larger number of deaths. The reason might be that citizens’ awareness of traffic safety is relatively weak, especially their ignorance of non-motor vehicle traffic safety. The non-MVAs risk also increased with the growing car ownership. It is worth noting that health input is positively correlated with the death toll of non-MVAs, which seems to be irrational and contrary to the research results of Wang et al. (2019). However, we thought this was a special point because a large amount of previous studies focused on the MVAs or all traffic fatalities, whereas we paid close attention to non-MVAs fatalities. On the one hand, when the motor vehicle and non-motor vehicle, or only non-motor vehicle suffered accidents, the outcomes of non-motor vehicle tend to be more serious for the lack of self-protection performance. On the other hand, the efficiency of rescue efforts may be slowed down by the possible uneven distribution of health personnel and inadequate emergency management mechanisms. In addition, economic development is positively correlated with the number of non-motor vehicle deaths, which is contrary to previous studies (Sun et al., 2019b). This also suggests that with the development of our country’s economy, to road traffic safety have been improved for the more construction investment and more effective policies implemented. While the road traffic accident problem is more complicated in recent years, so the traditional way may not reduce the death toll of non-MVAs effectively. The risk and outcome of accidents of the road traffic system will be changed by the change in the economy, population, road, vehicle, and other factors, so it is more necessary to improve interventions to cope with new models and ensure road traffic safety.

Several limitations of our study should be considered when interpreting our findings. Firstly, our data comes from police traffic accident records, which might be biased by under-reporting. Secondly, our data were collected in a retrospective cross-sectional study and could not be used to calculate the incidence and mortality of RTIs. Thirdly, some detailed information on RTIs was not available in public traffic accident statistics. In addition, there is a certain relationship among partial factors in the multiple linear regression analysis, which does not affect the use-value of the model, but the results should be carefully extrapolated. Finally, there are ecological fallacies in this study, and there may be research results inconsistent with the real situation. More studies are needed to enrich this topic. Despite these limitations, our study provides the trends of the last decade and possible influencing factors for RTIs in China based on available data and analysis.

This study provides the influencing factors and change trends of road traffic injuries in China in the recent 10 years, which can be used to advocate the improvement of road traffic safety in China. It is essential to draw up evidence-based interventions to control and promote safety in China. At first, it is of great importance to strengthen the safety education of non-motor vehicles, pedestrians and passengers without relaxing the intervention of MVAs, improve facilities and the quality of the driving environment, and identify high accidents for targeted management. In addition, it is necessary to to formulate and implement speed limit laws and regulations, improve roads through the state, apply new technologies and other means on vehicles to assist with speed limit, and develop an organized comprehensive pre-hospital and hospital emergency system. Last but not the least, researchers should continue to make efforts to improve the completeness and timeliness of RTI data in China, strive to make future research more reliable and comprehensive in description and prediction, and strengthen the collection of detailed data on non-MVAs, which encompasses road type, helmet wearing, accident severity and disease burden, in a word, we are committed to translating epidemiological results into preventive practices to reduce the disease burden of RTIs as well as the total injuries.

## Conclusion

RTIs in China showed different trends in the last decade, and the problem of non-motor vehicle traffic injuries has been neglected which need to be paid more attention. Moreover, considered the new changing trends and new traffic conditions, it is essential to adopt some targeted and effective intervention measures from other developed countries where the road traffic fatalities declined successfully and establish a comprehensive safety system to reduce RTIs in China.

## Supplemental Information

10.7717/peerj.14046/supp-1 Supplemental Information 1 Total Raw Data for Road Traffic Injuries.

## Additional Information and Declarations

### Data Availability

The following information was supplied regarding data availability: The raw measurements are available as a Supplemental File.
